# Predictive Value of the Systemic Immune-Inflammation Index for Cardiovascular Events in Patients With Chronic Total Coronary Occlusion

**DOI:** 10.31083/RCM52837

**Published:** 2026-09-18

**Authors:** Yuhao Zhao, Ping Wang, Shun Zhao, Qin Ma, Ze Zheng, Yuchen Shi, Jinghua Liu

**Affiliations:** ^1^Center for Coronary Artery Disease (CCAD), Beijing Anzhen Hospital, Capital Medical University, 100029 Beijing, China

**Keywords:** coronary chronic total occlusion, systemic immune-inflammation index, percutaneous coronary intervention, major adverse cardiovascular events

## Abstract

**Background:**

This large-scale cohort study aimed to evaluate the prognostic role of the systemic immune-inflammation index (SII) in patients with chronic total occlusion (CTO) undergoing percutaneous coronary intervention (PCI).

**Methods:**

A total of 3107 patients with CTO lesions who underwent PCI were enrolled in this study. Clinical characteristics were collected, and post-discharge major adverse cardiovascular events (MACEs) were recorded during follow-up, including all-cause mortality, non-fatal myocardial infarction, unplanned revascularization, and heart failure-related readmission. Patients were stratified into three groups according to SII tertiles: the low SII group (SII ≤447.66, n = 1036), the intermediate SII group (447.66 < SII < 679.89, n = 1039), and the high SII group (SII ≥679.89, n = 1032).

**Results:**

Over a median follow-up of 2.58 (2.33–2.92) years, 356 (11.5%) MACEs occurred, including 49 (1.6%) all-cause deaths, 105 (3.4%) non-fatal myocardial infarctions, 164 (5.3%) revascularizations, and 38 (1.2%) heart failure rehospitalizations. Multivariable Cox regression analyses demonstrated that elevated SII was independently linked to higher risks of MACEs (hazard ratio [HR] = 1.51, 95% confidence interval [CI]: 1.20–1.90; *p* = 0.001), all-cause death (HR = 2.06, 95% CI: 1.11–3.84; *p* = 0.022), non-fatal myocardial infarction (HR = 1.76, 95% CI: 1.15–2.69; *p* = 0.009), and repeat revascularization (HR = 1.32, 95% CI: 1.01–1.76; *p* = 0.046) after full adjustment. Kaplan–Meier survival curves showed significantly higher rates of adverse events in patients with elevated SII. Adding SII to traditional risk models improved predictive performance, as reflected by increases in the concordance index (C-index), net reclassification improvement (NRI), and integrated discrimination improvement (IDI).

**Conclusions:**

Baseline SII is a reliable and independent predictor of MACEs in patients with CTO after PCI, with superior predictive performance compared with conventional inflammatory markers.

## 1. Introduction

Coronary artery disease (CAD) remains a leading cause of mortality worldwide and is the most common cause of death globally [1]. Chronic total occlusion (CTO), a severe and complex subtype of CAD, is identified in approximately 20% of patients undergoing coronary angiography [2]. Advances in interventional devices and techniques have markedly improved the success rate of percutaneous coronary intervention (PCI) for CTO lesions [3]. Successful revascularization effectively relieves angina and reduces the burden of myocardial ischemia [4,5]. Nevertheless, the 10-year cardiac mortality rate remains as high as 10% even after successful CTO PCI, highlighting the urgent need to identify high-risk patients for early intensive intervention [6].

Atherosclerosis is now recognized as both a disease characterized by cholesterol accumulation within the vascular wall and as a systemic, chronic inflammatory vascular disorder [7]. Previous studies have demonstrated a close association between immune responses and inflammatory processes in the development of atherosclerosis [8,9]. A range of immune cells, including neutrophils, lymphocytes, monocytes, and platelets, participate in every stage of atherosclerotic plaque formation, progression, and rupture. For example, neutrophils accelerate atherosclerosis by activating macrophages and recruiting monocytes, whereas lymphocytes exert anti-atherosclerotic effects by modulating inflammatory responses [10,11]. In the pathophysiology of atherosclerosis, platelets contribute to both acute thrombosis and chronic vascular inflammation, which, in turn, promote the destabilization of advanced atherosclerotic plaques [12,13].

Conventional hematological inflammatory markers, including the neutrophil-to-lymphocyte ratio (NLR) and platelet-to-lymphocyte ratio (PLR), have been validated as predictors of CAD severity and prognosis in post-PCI patients [14,15]. Recently, the systemic immune-inflammation index (SII), defined as platelet count × neutrophil-to-lymphocyte ratio, has been identified as an integrated inflammatory biomarker associated with adverse cardiovascular outcomes in patients with coronary artery disease [16]. However, evidence regarding the prognostic value of SII specifically in patients with CTO remains limited. Therefore, this study was designed to investigate whether SII can predict long-term cardiovascular events in patients with CTO following PCI.

## 2. Materials and Methods

### 2.1 Subjects

This single-center retrospective observational study was conducted at the Heart Center of Beijing Anzhen Hospital, affiliated with Capital Medical University. A total of 3107 patients who met the inclusion criteria were admitted between January 2019 and November 2021 and were enrolled in this study. In addition, 458 patients with non-CTO lesions served as the control group.

The inclusion criteria were as follows: age >18 years and angiographically confirmed CTO, defined as Thrombolysis in Myocardial Infarction (TIMI) flow grade 0 with an occlusion duration ≥3 months [17]. The indication for PCI in patients with CTO was based on a clinical assessment of symptomatic relief and ischemic burden. All patients included in the study had either refractory angina despite optimal medical therapy or evidence of significant ischemia (≥10% myocardial ischemia by stress imaging or Fractional Flow Reserve (FFR) <0.80 in the CTO-related territory) [18]. For subjects with multivessel coronary artery disease or a history of multiple interventional procedures, only the initial CTO PCI procedure was recorded.

In this study, FFR measurements in the CTO-related territory were obtained after successful lesion crossing and revascularization. A pressure-sensing guidewire was advanced into the distal vessel, and baseline pressures (Pd/Pa) were recorded. Maximal hyperemia was induced using intravenous adenosine (140 μg/kg/min) or intracoronary adenosine (100–200 μg). The FFR value, calculated as Pd/Pa during hyperemia, was displayed by the monitoring system. An FFR <0.80 indicated significant ischemia, aiding in the assessment of the functional significance of the CTO and confirming the adequacy of revascularization.

The exclusion criteria were as follows: acute or chronic infectious or inflammatory diseases; autoimmune diseases; acute coronary syndrome within 1 month; severe heart failure (left ventricular ejection fraction [LVEF] <30%) or cardiogenic shock; malignant tumors; severe hepatic or renal dysfunction; use of glucocorticoids or immunosuppressants; incomplete clinical data. In addition, all control patients met the above exclusion criteria to reduce selection bias. All enrolled participants provided written informed consent, and the study protocol was approved by the institutional ethics committee in accordance with the Declaration of Helsinki.

### 2.2 Laboratory Measurements

Baseline clinical indicators, including demographic characteristics and medical history, were recorded at study enrollment. After admission, fasting venous blood samples were collected from all patients for laboratory testing, including white blood cell (WBC), neutrophil, lymphocyte, and platelet counts; hemoglobin; triglycerides; total cholesterol (TC); high-density lipoprotein cholesterol (HDL-C); low-density lipoprotein cholesterol (LDL-C); high-sensitivity C-reactive protein (hs-CRP); and other laboratory parameters.

The SII was calculated as follows: SII = platelet count × neutrophil count/lymphocyte count (×10^9^/L). Meanwhile, NLR and PLR were also calculated for comparative analysis.

### 2.3 Angiography and Endpoints

Procedural success of CTO intervention was defined as complete revascularization of the target chronic total occlusion, residual stenosis <30% in the lesion segments, and restoration of TIMI grade 3 coronary blood flow. In addition, no in-hospital major adverse cardiovascular events (MACEs) occurred during hospitalization, including all-cause death, myocardial infarction, clinically indicated target vessel revascularization by PCI or coronary artery bypass surgery, cardiac tamponade requiring intervention or surgery, and ischemic or hemorrhagic stroke.

Two widely adopted scoring systems were used to assess the morphological complexity of CTO lesions. The J-CTO score includes five core variables: stump morphology, lesion calcification, vessel bending >45 degrees, lesion length >20 mm, and prior failed intervention attempts. The PROGRESS CTO score primarily evaluates proximal cap ambiguity, collateral circulation, vessel tortuosity, and lesion location in the circumflex artery.

Long-term follow-up data were obtained through telephone interviews, electronic medical record review, and regular outpatient reexamination. The primary endpoint was MACEs, defined as a composite of all-cause death, non-fatal MI, unplanned revascularization, and rehospitalization for heart failure. The diagnostic criteria for myocardial infarction were based on the fourth universal definition [19]. Unplanned revascularization events were defined as any subsequent unexpected PCI or emergency coronary artery bypass grafting (CABG).

### 2.4 Statistical Analysis

Categorical variables are expressed as counts and percentages. Continuous variables are presented as the mean ± standard deviation (SD) or median with interquartile range (IQR, 25th–75th percentiles). Normality was assessed using the Kolmogorov–Smirnov test. Continuous variables were compared using the Student’s *t*-test for parametric data and the Mann–Whitney U test for nonparametric data. The Kruskal–Wallis test was used to compare three or more cohorts. The chi-square test was applied for categorical variables, and Fisher’s exact test was applied when appropriate.

The Kaplan–Meier method was used to analyze long-term prognosis and cumulative event rates across SII subgroups, with intergroup differences assessed using the log-rank test. Receiver operating characteristic (ROC) curve analysis was conducted to compare the predictive performance of SII, NLR, PLR, hs‑CRP, and WBC for MACEs, with the DeLong test used to assess pairwise differences among these curves.

Multivariable Cox regression models were constructed to assess the independent association between SII and adverse cardiovascular outcomes. Three adjusted models were established: Model 1 included age, body mass index (BMI), and sex; Model 2 additionally included smoking status, hypertension, diabetes, prior myocardial infarction, and history of PCI; Model 3 further adjusted for TC, LDL-C, LVEF, and procedural success of CTO recanalization, with stepwise regression used for variable screening. Stratified sensitivity analyses were conducted based on CTO PCI procedural results and SII levels. We computed the C-index, net reclassification improvement (NRI), and integrated discrimination improvement (IDI) to evaluate whether adding SII to traditional risk models improved predictive accuracy for adverse cardiovascular events. All statistical analyses were performed using IBM SPSS Statistics 24.0 (IBM, Armonk, NY, USA) and R 4.4 (R Foundation for Statistical Computing, Vienna, Austria). A two-tailed *p*-value < 0.05 was considered statistically significant.

## 3. Results

### 3.1 Baseline Characteristics

The median SII level, with interquartile ranges, was markedly elevated in patients with CTO lesions relative to those without CTO: 551.11 (397.54–776.20) vs. 453.20 (347.46–673.85), respectively (*p* < 0.001). Moreover, patients with unsuccessful CTO PCI had a significantly higher SII than the overall CTO cohort, with a median of 667.31 (447.84–964.24) compared with 551.11 (397.54–776.20) (*p* < 0.0001; Fig. 1).

**Fig. 1.**
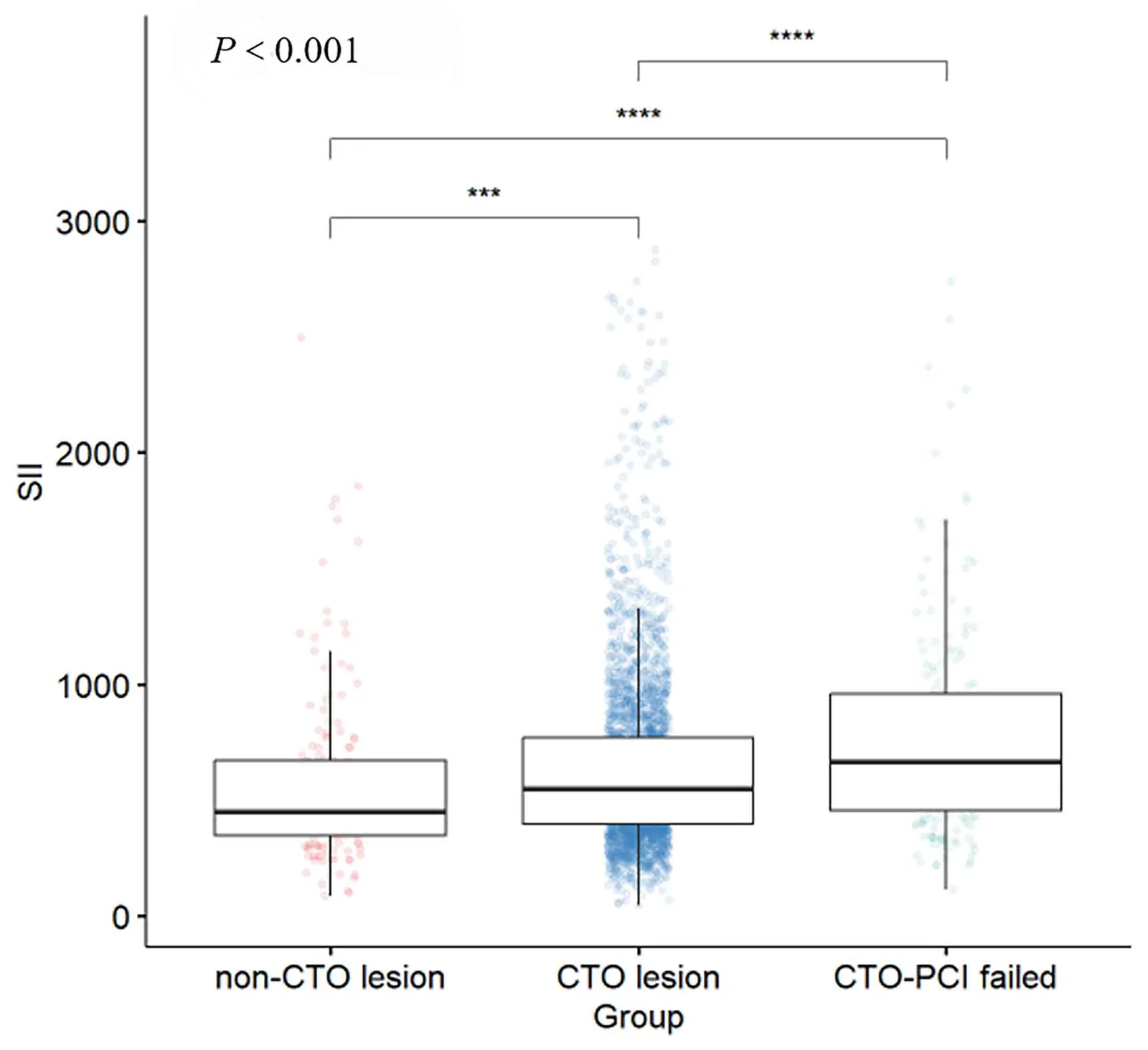
**SII levels in patients with non-CTO lesions, CTO lesions, and failed CTO PCI**. SII, systemic immune-inflammation index; CTO, chronic total occlusion; PCI, percutaneous coronary intervention. Note: *** *p* < 0.001; **** *p* < 0.0001.

Since SII levels were not normally distributed, the 3107 patients with CTO lesions were stratified into three subgroups based on the SII tertiles: the low SII group (SII ≤447.66, n = 1036), the middle SII group (447.66 < SII < 679.89, n = 1039), and the high SII group (SII ≥679.89, n = 1032). The demographic, clinical, and laboratory characteristics of all enrolled patients are summarized in Table 1. Among the study population, 81.8% were male, and the median age was 60 years (52–67). Regarding demographic and clinical characteristics, significant differences were observed among the three subgroups in the prevalence of hypertension, diabetes mellitus, and stroke. In terms of laboratory parameters, patients in the high SII group exhibited higher WBC, neutrophil, platelet, and monocyte counts, as well as higher PLR, NLR, D-dimer, and hs-CRP levels. In contrast, these indicators had lower lymphocyte and hemoglobin counts. Nevertheless, J-CTO and PROGRESS CTO scores did not differ significantly among the three groups. Echocardiographic evaluation of cardiac remodeling showed no significant correlations between SII levels and left ventricular end-diastolic diameter (LVEDD), left ventricular end-systolic diameter (LVESD), or LVEF. Although RCA-CTO PCI was the most common intervention among all patients, the difference was not statistically significant (*p* = 0.706). The overall CTO PCI procedural success rate reached 82.7%, with the high SII group exhibiting notably lower success rates than the other two groups.

**Table 1.** **Baseline clinical and laboratory characteristics of patients with CTO according to SII group**.

		Overall (n = 3107)	Tertiles of the systemic immune-inflammation index			*p*-value
			Lower group (n = 1036)	Mid group (n = 1039)	Higher group (n = 1032)	
Demographics						
	Age, years	60 (52, 67)	60 (53, 66)	59 (52, 66)	60 (53, 68)	0.166
	Male, n (%)	2541 (81.8)	860 (83.0)	860 (82.8)	821 (79.6)	0.075
	BMI, kg/m^2^	26.2 ± 4.3	26.2 ± 3.5	26.3 ± 3.4	26.1 ± 5.2	0.428
	Current smoker, n (%)	1004 (32.4)	344 (33.3)	345 (33.4)	315 (30.6)	0.325
	Previous stroke, n (%)	223 (7.2)	59 (5.7)	75 (7.2)	89 (8.6)	0.036
	Diabetes, n (%)	1318 (42.4)	408 (39.4)	437 (42.1)	473 (45.8)	0.012
	Hypertension, n (%)	2087 (67.2)	644 (62.2)	695 (66.9)	748 (72.5)	<0.001
	Previous PCI, n (%)	1313 (42.3)	431 (41.6)	433 (41.7)	449 (43.5)	0.610
	Previous MI, n (%)	730 (23.5)	239 (23.1)	240 (23.1)	251 (24.3)	0.746
Laboratory findings						
	WBC, ×10^9^/L	6.75 (5.69, 8.00)	6.22 (5.29, 7.34)	6.77 (5.73, 7.84)	7.41 (6.25, 8.72)	<0.001
	Lymphocyte, ×10^9^/L	1.59 (1.17, 2.06)	1.88 (1.45, 2.36)	1.63 (1.28, 2.04)	1.30 (0.96, 1.68)	<0.001
	Neutrophil, ×10^9^/L	4.36 (3.51, 5.34)	3.55 (2.97, 4.23)	4.43 (3.73, 5.10)	5.37 (4.39, 6.54)	<0.001
	Monocyte, ×10^9^/L	0.40 (0.32, 0.50)	0.39 (0.31, 0.47)	0.40 (0.32, 0.50)	0.42 (0.34, 0.54)	<0.001
	Platelets, ×10^9^/L	211 (178, 251)	186 (155, 215)	211 (182, 247)	241 (204, 282)	<0.001
	NLR	2.59 (1.98, 3.56)	1.82 (1.49, 2.16)	2.56 (2.21, 3.06)	3.99 (3.2, 5.1)	<0.001
	PLR	125 (97, 164)	93 (76, 113)	125 (106, 147)	177 (144, 224)	<0.001
	hs-CRP, mg/L	1.13 (0.58, 2.47)	0.96 (0.51, 1.96)	1.14 (0.60, 2.59)	1.39 (0.65, 3.12)	<0.001
	Hemoglobin, g/L	143 (132, 152)	144 (133, 153)	143 (133, 154)	141 (130, 150)	<0.001
	Creatinine, μmol/L	73.9 (64.4, 84.8)	73.4 (65.2, 84.0)	74.6 (64.3, 83.7)	74.4 (64.0, 85.9)	0.740
	Triglycerides, mmol/L	1.50 (1.11, 2.08)	1.51 (1.11, 2.13)	1.47 (1.09, 2.06)	1.51 (1.12, 2.10)	0.594
	TC, mmol/L	3.67 (3.13, 4.40)	3.65 (3.09, 4.42)	3.69 (3.13, 4.43)	3.68 (3.14, 4.38)	0.639
	HDL-C, mmol/L	0.94 (0.81, 1.10)	0.94 (0.80, 1.10)	0.94 (0.83, 1.09)	0.95 (0.82, 1.10)	0.556
	LDL-C, mmol/L	2.00 (1.55, 2.58)	1.96 (1.52, 2.58)	2.03 (1.55, 2.60)	2.03 (1.58, 2.57)	0.336
	BNP, pg/mL	54 (28, 108)	54 (31, 119)	51 (28, 106)	59 (27, 138)	0.066
	D-dimer, ng/mL	89 (56, 140)	86 (54, 133)	90 (57, 138)	94 (59, 148)	0.012
	J-CTO score	2 (1, 3)	2 (1, 3)	2 (1, 3)	2 (1, 3)	0.919
	PROGRESS score	1 (0, 2)	1 (1, 2)	1 (1, 2)	1 (0, 2)	0.807
Echocardiography						
	LVEDD, mm	48.9 ± 5.66	49.1 ± 5.77	48.8 ± 5.60	48.7 ± 5.60	0.633
	LVESD, mm	32.6 ± 3.44	33.1 ± 6.7	32.5 ± 6.4	32.3 ± 6.0	0.356
	LVEF, %	59.7 ± 8.8	59.2 ± 9.2	59.5 ± 9.1	60.2 ± 8.6	0.221
Medications, n (%)						
	DAPT	3088 (99.4)	1031 (99.5)	1033 (99.4)	1024 (99.2)	>0.999
	Statin	3074 (98.9)	1025 (98.9)	1024 (98.6)	1025 (99.3)	>0.999
	β-blockers	2252 (72.5)	752 (72.6)	756 (72.8)	744 (72.1)	0.938
	ACE inhibitor/ARB	1780 (57.3)	591 (57.0)	580 (55.8)	609 (59.0)	0.577
	CCBs	1015 (32.7)	330 (31.9)	340 (32.7)	345 (33.4)	0.757
	Nitrates	786 (25.3)	265 (25.6)	263 (25.3)	258 (25.0)	0.989
Treatment of CTO lesions, n (%)						0.706
	LAD	1140 (36.7)	363 (35.0)	391 (37.6)	386 (37.4)	-
	LCX	636 (20.5)	214 (20.7)	215 (20.7)	207 (20.1)	-
	RCA	1331 (42.8)	459 (44.3)	433 (41.7)	439 (42.5)	-
	Successful CTO PCI	2570 (82.7)	883 (85.2)	885 (85.2)	802 (77.7)	0.009

MACEs, major adverse cardiovascular events; SII, systemic immune-inflammation index; BMI, body mass index; MI, myocardial infarction; WBC, white blood cell count; PLR, platelet-to-lymphocyte ratio; NLR, neutrophil-to-lymphocyte ratio; hs-CRP, high-sensitivity C-reactive protein; BNP, brain natriuretic peptide; TC, total cholesterol; LDL-C, low-density lipoprotein cholesterol; HDL-C, high-density lipoprotein cholesterol; LVESD, left ventricular end-systolic diameter; LVEDD, left ventricular end-diastolic diameter; LVEF, left ventricular ejection fraction; DAPT, dual antiplatelet therapy; ACE, angiotensin-converting enzyme; ARB, angiotensin II receptor blocker; CCB, calcium channel blocker; LAD, left anterior descending artery; LCX, left circumflex artery; RCA, right coronary artery.

### 3.2 Association Between SII and Outcomes in Patients With CTO

During a median follow-up of 2.58 (2.33–2.92) years, 356 MACEs occurred, accounting for 11.5% of the total study population. These events included 49 cases (1.6%) of all-cause death, 105 cases (3.4%) of non-fatal myocardial infarction (MI), 164 cases (5.3%) of revascularization, and 38 cases (1.2%) of heart failure hospitalization (Table 2). Among patients with higher baseline SII levels, 162 (15.7%) experienced MACEs, compared with 111 (10.7%) in the middle SII group and 83 (8.0%) in the low SII group, with significant intergroup differences (*p* < 0.001). Nevertheless, no significant difference in heart failure-related readmission rates was detected across the three subgroups (*p* = 0.826). Kaplan–Meier analysis revealed that patients with low-to-moderate SII exhibited significantly lower long-term risks of MACE, all-cause mortality, non-fatal MI, and revascularization than those with elevated SII values (Fig. 2). These findings indicate that elevated SII levels are correlated with an increased risk of cardiovascular events.

**Table 2.** **Clinical outcomes in patients with CTO based on SII scoring groups**.

	Overall (n = 3107)	Tertiles of the systemic immune-inflammation index			*p*-value
		Lower group (n = 1036)	Mid group (n = 1039)	Higher group (n = 1032)	
MACEs, n (%)	356 (11.5)	83 (8.0)	111 (10.7)	162 (15.7)	<0.001
All-cause death, n (%)	49 (1.6)	10 (1.0)	15 (1.4)	24 (2.3)	0.042
Non-fatal MI, n (%)	105 (3.4)	20 (1.9)	29 (2.8)	56 (5.4)	<0.001
Revascularization, n (%)	164 (5.3)	42 (4.1)	54 (5.2)	68 (6.6)	0.036
Congestive HF, n (%)	38 (1.2)	11 (1.1)	13 (1.3)	14 (1.4)	0.826

HF, heart failure.

**Fig. 2.**
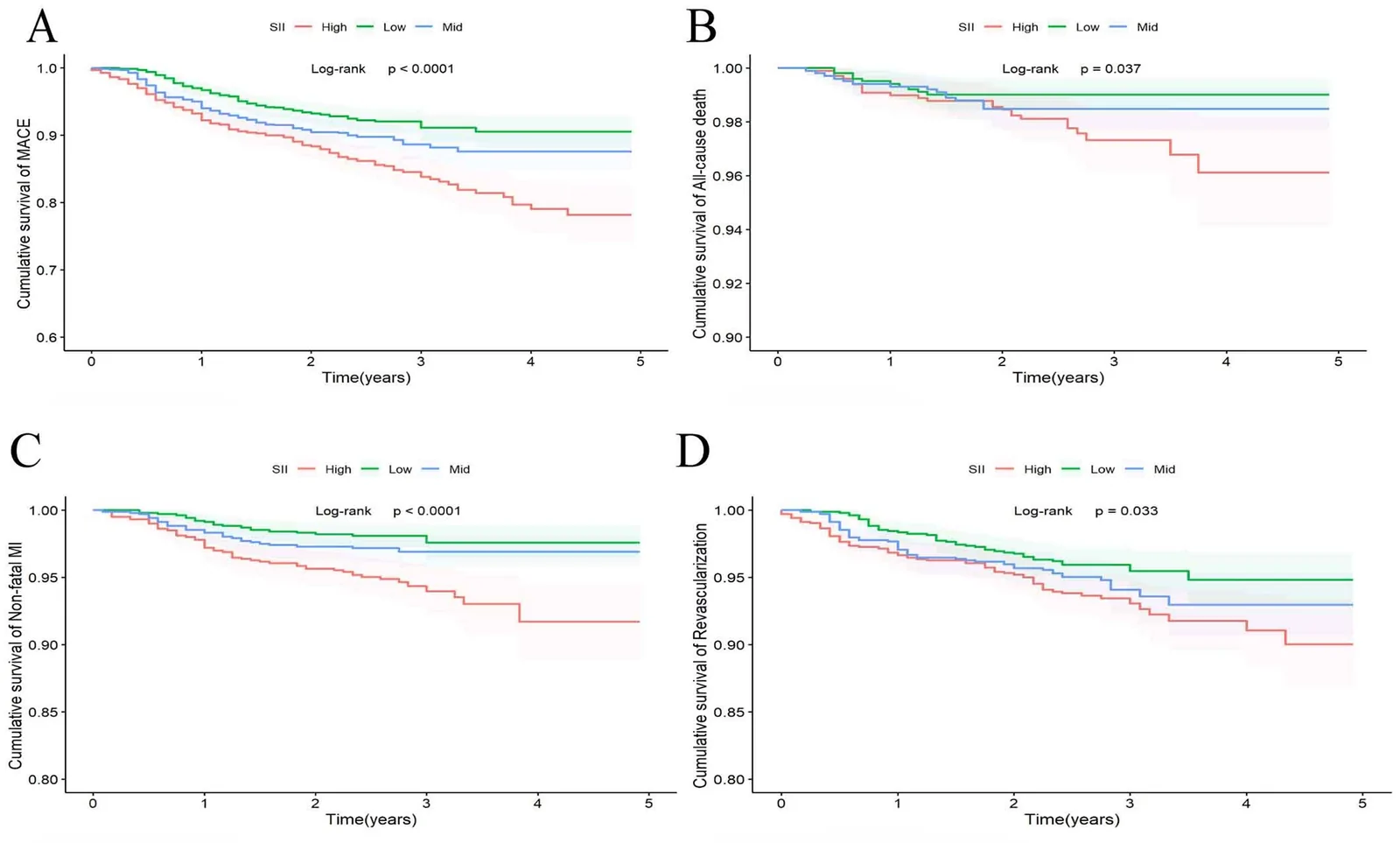
**Kaplan–Meier survival curve analysis of (A) MACEs, (B) all-cause mortality, (C) non-fatal MI, and (D) revascularization**.

ROC curves were used to assess the incremental predictive value of SII for MACEs beyond that of NLR, PLR, WBC count, and hs-CRP level (Fig. 3). The area under the curve (AUC) for SII in predicting MACEs was higher than that for NLR (AUC: 0.67, 95% confidence interval (CI): 0.64–0.70 vs. AUC: 0.65, 95% CI: 0.62–0.69; *p* = 0.019), PLR (AUC: 0.63, 95% CI: 0.60–0.67; *p* = 0.003), hs-CRP level (AUC: 0.53, 95% CI: 0.49–0.56; *p* < 0.001), and WBC count (AUC: 0.52, 95% CI: 0.49–0.55; *p* < 0.001). These findings indicate that SII outperforms NLR, PLR, hs-CRP, and WBC count in predicting MACEs.

**Fig. 3.**
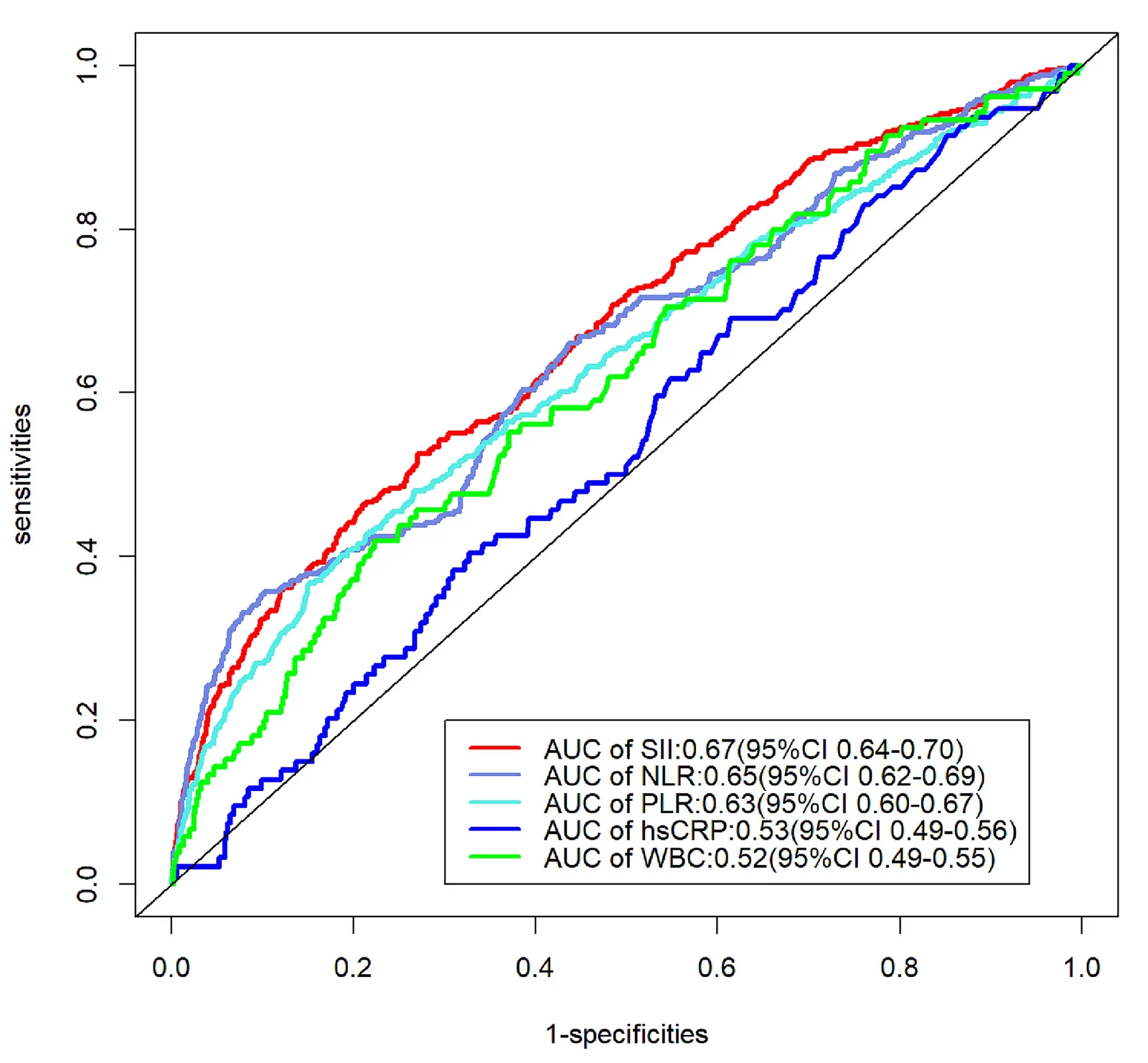
**Receiver operating characteristic (ROC) curve analysis of the inflammatory index for predicting MACEs**.

Cox proportional hazards regression analysis demonstrated that, after adjusting for age, sex, BMI, current smoking status, hypertension, diabetes, previous MI, previous PCI, LDL-C, TC, LVEF, and successful CTO treatment, higher SII was associated with an increased risk of future MACEs (HR: 1.51, 95% CI: 1.20–1.90; *p* = 0.001), all-cause mortality (HR: 2.06, 95% CI: 1.11–3.84; *p* = 0.022), non-fatal MI (HR: 1.76, 95% CI: 1.15–2.69; *p* = 0.009), and repeated revascularization (HR: 1.32, 95% CI: 1.01–1.76; *p* = 0.046) in multivariable Model 3 (Table 3). Furthermore, a significant association between SII and MACEs was observed in multivariable Models 1 and 2. We conducted a sensitivity analysis to investigate the impact of successful CTO PCI on the relationship between SII and MACEs. The prognostic significance of SII in patients with successful CTO PCI was closely consistent with the primary analysis (middle SII (M-SII) vs. low SII (L-SII): HR 1.32, 95% CI 1.11–1.57; *p* = 0.012; high SII (H-SII) vs. low SII (L-SII): HR 2.32, 95% CI 1.56–3.12; *p* < 0.001), whereas no such significant association was found in patients with failed CTO PCI (Table 4).

**Table 3.** **Association between SII and adverse events in patients with CTO after PCI**.

	Model 1^a^		Model 2^b^		Model 3^c^	
	HR (95% CI)	*p*-value	HR (95% CI)	*p*-value	HR (95% CI)	*p*-value
MACEs	1.69 (1.37–2.09)	<0.001	1.59 (1.23–1.96)	<0.001	1.51 (1.20–1.90)	0.001
All-cause death	1.89 (1.06–3.34)	0.031	1.96 (1.09–3.50)	0.024	2.06 (1.11–3.84)	0.022
Non-fatal MI	2.28 (1.55–3.34)	<0.001	2.04 (1.38–3.00)	<0.001	1.76 (1.15–2.69)	0.009
Revascularization	1.44 (1.09–1.96)	0.022	1.38 (1.06–1.88)	0.042	1.32 (1.01–1.76)	0.046

^a^Model 1: adjusted for age, sex, and BMI; ^b^Model 2: adjusted for age, sex, BMI, current smoker, diabetes, hypertension, previous MI, and previous PCI; ^c^Model 3: adjusted for age, sex, BMI, current smoker, diabetes, hypertension, previous MI, previous PCI, LDL-C, TC, LVEF, and successful CTO treatment. CI, confidence interval; HR, hazard ratio.

**Table 4.** **Sensitivity analysis of the relationship between SII and MACEs based on PCI outcomes for CTO lesions**.

	Lower group		Mid group		Higher group	
	HR (95% CI)	*p*-value	HR (95% CI)	*p*-value	HR (95% CI)	*p*-value
Successful CTO PCI	1 (reference)	-	1.32 (1.11–1.57)	0.012	2.32 (1.56–3.12)	<0.001
Failed CTO PCI	1 (reference)	-	1.02 (0.89–1.42)	0.259	1.43 (0.52–2.67)	0.538

Adjusted for age, BMI, sex, smoking status, diabetes, hypertension, previous MI, previous PCI, LDL-C, TC, and LVEF.

### 3.3 Incorporation of the SII Into Baseline Models for Outcome Prediction in CTO Patients

Adding SII to the baseline model significantly improved predictive performance for MACEs (*p* < 0.001), all-cause death (*p* = 0.010), non-fatal MI (*p* < 0.001), and revascularization (*p* = 0.007), as demonstrated by a significant increase in the C-index (Table 5). Reclassification analysis further showed that incorporating SII resulted in an NRI of 0.23 (*p* < 0.001) and an IDI of 0.06 (*p* = 0.001) for MACEs. For all-cause death, the NRI was 0.09(*p* = 0.019), and the IDI was 0.03 (*p* = 0.008). For non-fatal MI, the NRI was 0.07 (*p* = 0.008), and the IDI was 0.04 (*p* < 0.001). For revascularization, the NRI was 0.13 (*p* = 0.011), and the IDI was 0.004 (*p* = 0.031) (Table 5). These results show that adding SII to prognostic models yields superior predictive performance compared with conventional risk factors alone in post-PCI CTO patients.

**Table 5.** **Performance assessment of novel prognostic models for adverse outcomes**.

		C-index (95% CI)	*p-*value	NRI (95% CI)	*p-*value	IDI (95% CI)	*p-*value
MACEs							
	Traditional risk factors	0.70 (0.67–0.73)	–	Ref	–	Ref	–
	New risk factors	0.76 (0.73–0.79)	<0.001	0.23 (0.08–0.31)	<0.001	0.06 (0.05–0.08)	0.001
All-cause mortality							
	Traditional risk factors	0.84 (0.78–0.89)	–	Ref	–	Ref	–
	New risk factors	0.86 (0.81–0.92)	0.010	0.09 (0.02–0.21)	0.019	0.03 (0.01–0.07)	0.008
Non-fatal MI							
	Traditional risk factors	0.86 (0.83–0.89)	–	Ref	–	Ref	–
	New risk factors	0.90 (0.87–0.92)	<0.001	0.07 (0.02–0.24)	0.008	0.04 (0.02–0.06)	<0.001
Revascularization							
	Traditional risk factors	0.61 (0.56–0.67)	–	Ref	–	Ref	–
	New risk factors	0.64 (0.59–0.68)	0.007	0.13 (0.04–0.23)	0.011	0.00 (0.00–0.01)	0.031

**Notes: **Traditional risk factors included age, sex, BMI, smoking status, diabetes, hypertension, and previous MI. IDI, integrated discrimination improvement; NRI, net reclassification improvement.

## 4. Discussion

To our knowledge, this is the first study to explore the association between baseline SII and the risk of cardiovascular events in patients with CTO lesions. Elevated SII may independently predict adverse outcomes in post-PCI CTO patients. In addition, compared with traditional indicators such as WBC, PLR, NLR, and hs-CRP, higher baseline SII levels appear to be more effective in predicting MACEs. Incorporating SII into the clinical predictive model enhanced the ability of the metric to predict MACEs in CTO patients after PCI, as evidenced by an improved C-index.

CTO PCI is considered one of the most challenging procedures in coronary interventional cardiology [2]. With continuous advances in CTO interventional techniques and evolving concepts, the success rate of this procedure has steadily increased [3]. Nevertheless, even after successful CTO PCI, the 10-year cardiac mortality rate remains 10% [6]. To alleviate the burden and damage caused by CTO lesions, it is necessary to explore the relationship between relevant indicators and CTO-related risks. This may facilitate the identification of high-risk patients with CTO who require early clinical intervention.

Atherosclerosis is the primary cause of CAD and is closely associated with persistent inflammatory responses [20]. The rupture of atherosclerotic plaques involves complex interactions between the innate and adaptive immune systems [21]. Neutrophils undergoing suicidal death can release pro-oxidative substances and pro-inflammatory mediators, which contribute to the formation of neutrophil extracellular traps (NETs). Notably, NETs exert multiple prothrombotic effects by facilitating platelet adhesion, activation, and aggregation, thereby inducing thrombosis and increasing the risk of acute complications, including MI [22,23]. As a marker of subclinical inflammation, NLR has been identified in prior studies as an independent predictor of cardiovascular events and mortality among patients with coronary artery disease, particularly those with ST-segment elevation myocardial infarction [24]. Additionally, NLR is closely correlated with the severity of coronary atherosclerosis and the morphological characteristics of atherosclerotic plaques [25]. In addition to NLR, experimental studies have demonstrated that platelets interact with neutrophils in key biological processes underlying atherosclerosis and thrombosis [26]. Intravascular platelet aggregation and endothelial damage are also crucial contributors to the development of atherosclerosis, which often leads to adverse cardiovascular events. As a reliable biomarker for CAD, platelets can effectively predict thrombotic potential and blood vulnerability [27]. It has also been reported that PLR is an effective predictor of severe atherosclerosis [28].

Recently, SII has emerged as a novel indicator based on circulating immune and inflammatory cells. SII integrates information from platelet, neutrophil, and lymphocyte counts and may reflect three key pathways: thrombosis, the inflammatory response, and the adaptive immune response [29]. This biomarker was originally proposed by Hu et al. [30], and existing research has shown that it effectively reflects the severity of systemic inflammation in cancer patients and has high prognostic value across different cancer types. Yang et al. [31] found that elevated SII independently correlates with cardiovascular events, including non-fatal myocardial infarction and stroke, and exhibits superior prognostic performance for MACEs relative to conventional inflammatory and hematologic markers. Liu et al. [32] demonstrated that SII outperforms PLR, NLR, and CRP in predicting the occurrence of coronary artery disease. Prior research identified elevated SII as an independent long-term prognostic marker for MACEs and mortality in patients with three-vessel coronary disease post-revascularization [33]. Another study showed that elevated SII is independently linked to ischemia in patients with non-obstructive coronary artery disease [34]. Notably, SII appears to be more stable than a single blood cell count, which can be easily affected by various interfering factors such as fluid overload and dehydration [35]. Another study demonstrated that SII levels were significantly higher in patients with ST-segment elevation myocardial infarction who died during in-hospital follow-up than in those who were discharged alive [36]. A recent meta-analysis further confirmed that SII has strong prognostic value for both MACEs and all-cause mortality in patients with acute coronary syndrome [37]. However, research exploring the relationship between cardiovascular risk and SII in patients with CTO lesions remains limited. One study revealed that elevated SII is strongly correlated with poor collateral circulation in CTO lesions and has superior risk-stratification capacity compared with individual inflammatory markers [38]. When the coronary artery is completely occluded, the pressure gradient within the artery and the shear stress on the arterial wall increase, leading to inflammation and oxidative reactions and stimulating the growth of coronary collateral vessels. During this process, neutrophils and lymphocytes activate a series of downstream molecular pathways and participate in the expression of monocyte attraction- and adhesion-related proteins [39]. In contrast, well-developed coronary collateral circulation can improve the quality of life and prognosis of patients with CTO [40]. In our study, baseline SII in the CTO group was significantly higher than that in the non-CTO group, indicating more severe systemic inflammation. Meanwhile, patients with higher SII levels had a lower rate of successful CTO PCI (77% vs. 85%). This difference in PCI success rates may be a confounding factor in clinical outcomes, as successful CTO PCI is known to significantly improve the prognosis of patients with CTO. The lower PCI success rate in the high SII group could partially explain their poorer clinical outcomes. In our sensitivity analysis, SII showed a significant correlation with cardiovascular risk only among patients who underwent successful CTO PCI; conversely, this association was not observed among patients with unsuccessful CTO revascularization. This suggests that successful CTO PCI may modify patient prognosis and highlights the dual role of SII. As a comprehensive marker of inflammation and immune status, SII reflects both the overall health status and PCI response capacity of patients, as well as their potential for recovery. For patients with failed CTO PCI, prognosis may be more strongly influenced by anatomical complexity and unresolved issues, such as persistent CTO lesions and ongoing blood flow disturbances.

Hypertension and diabetes are important precursors of cardiovascular disease. The “common soil” hypothesis posits that cardiovascular diseases and diabetes share common genetic and environmental factors, with chronic low-grade inflammation serving as a key driver of their pathogenesis [41]. Prior research identified a stronger positive association between SII and coronary artery disease among patients with diabetes or hypertension [42]. This may be attributable to inflammatory infiltration, endothelial dysfunction, and vascular remodeling induced by chronic inflammation in these patients [43]. These results may indicate a unique metabolic profile in patients with diabetes compared with the general population. In addition, compared with the low and medium SII groups, the high SII group had a higher proportion of patients with hypertension and diabetes at baseline (72.5% and 45.8%, respectively), which may partly explain the association between SII and the higher incidence of cardiovascular events. This study further expands the clinical significance of SII in cardiovascular disease. In our analysis, the AUC values for all inflammatory indices, including SII, were relatively low. This may be related to the baseline characteristics of the specific patient population, individual differences in inflammatory responses, or other confounding clinical factors. In the current study, incorporating SII into clinical prediction models both enhanced the predictive ability for MACEs in patients with CTO and reclassified patients into different risk categories, as indicated by NRI and IDI analyses. These findings suggest that elevated SII may independently predict subsequent adverse cardiovascular events and improve prognostic performance for adverse outcomes in patients with CTO.

The effect of coronary revascularization for stable angina remains unclear, especially when pharmacological therapies can effectively control symptoms. While revascularization has been shown to significantly improve symptoms and reduce cardiovascular events in certain patients, not all patients benefit from coronary revascularization. SII assessment has the potential to identify patients who may benefit from revascularization. In patients with stable angina, elevated SII levels may reflect increased inflammation, endothelial dysfunction, and progression of atherosclerosis. These factors could indicate a higher risk of cardiovascular events and may help guide the decision to proceed with coronary revascularization. Specifically, SII assessment could aid patient selection by identifying those at higher risk of adverse outcomes. Higher SII levels may indicate a greater inflammatory burden that could be mitigated by coronary revascularization through improved blood flow, symptom relief, and reduced risk of future cardiovascular events. Conversely, patients with lower SII levels might be adequately managed with medical therapy alone, potentially avoiding unnecessary interventions. For instance, if a significant decrease in SII is observed post-PCI, it may suggest that the procedure was successful and has helped reduce thrombus formation and the progression of atherosclerosis. This change could serve as a useful indicator of treatment success and inform further therapeutic decisions. While further clinical validation is needed, SII as a composite marker offers a novel approach to identifying patients with stable angina who may benefit from coronary revascularization.

The interactions among neutrophils, lymphocytes, and platelets may provide new therapeutic targets to alleviate chronic inflammation, which is often associated with MI. One study emphasized that anti-inflammatory interventions may slow atherosclerotic progression and lower the risk of adverse cardiovascular events [44]. The large-scale randomized, double-blind Canakinumab Anti-Inflammatory Thrombosis Outcomes Study (CANTOS) showed that targeted inhibition of IL-1β lowers hs-CRP concentrations and reduces the risk of cardiovascular events. Notably, there was no significant reduction in lipid levels in the experimental group after treatment. As the first trial to confirm that reducing inflammation can reduce adverse cardiovascular events independently of lipid levels, CANTOS provides strong evidence that targeted anti-inflammatory treatment is effective in managing atherosclerosis [45]. In addition, colchicine exerts anti-inflammatory effects by inhibiting microtubule proteins, thereby suppressing microtubule aggregation, neutrophil activation, and migration. The Colchicine Cardiovascular Outcomes Trial (COLCOT) demonstrated that colchicine therapy decreases recurrent cardiovascular events in patients following acute myocardial infarction. Simultaneously, supplementary experiments indicated that patients in the colchicine treatment group had smaller infarct sizes and lower inflammatory marker levels, including neutrophil counts [46]. SII reflects the systemic immune-inflammatory state, which may explain its association with MACEs. For individuals with elevated SII levels detected during routine physical examinations, lifestyle interventions to reduce inflammation may be recommended [47]. In addition, for the general population with high SII levels, it may be necessary to increase the frequency of medical examinations appropriately to facilitate early detection and intervention, thereby improving clinical outcomes.

The SII index, which requires only a complete blood count, is an attractive option for reflecting systemic inflammation levels due to its high availability and low cost. In conclusion, our study suggests that SII may help identify high-risk CTO patients who warrant intensified therapeutic interventions and closer follow-up.

## 5. Limitations

This study has several limitations. First, the study is retrospective, which limits the ability to establish causal relationships and may introduce potential bias. The causes of death were not reported, which could limit our understanding of the factors contributing to patient outcomes. Additionally, since the study population consisted predominantly of Asian male patients, the generalizability of the findings to non-Asian populations and women may be limited. Further prospective, multicenter studies with diverse ethnic populations, balanced sex representation, and complete mortality data are needed to validate and extend these findings. Second, SII and other inflammatory markers were measured only at baseline, without serial assessments during follow-up. Third, although multivariable analytical methods were employed, unmeasured confounding factors may still have influenced the outcomes. Inflammatory conditions, such as obesity and metabolic syndrome, may affect SII, and subgroup analyses are needed for further investigation. Fourth, collateral circulation in CTO patients was not recorded in this study database. Future studies could combine collateral circulation status with CTO treatment response (*e*.*g*., PCI success rate, incidence of cardiovascular events) to further explore how collateral circulation affects treatment response. Finally, it remains unclear whether drugs such as aspirin or statins can reduce the risks associated with inflammatory conditions.

## 6. Conclusions

Based on the above results, the baseline SII appears to be a reliable biomarker in CTO patients undergoing PCI; compared with traditional indicators, baseline SII may have better predictive ability for MACEs. Our findings further advance the understanding of SII as a valuable risk-stratification tool for cardiovascular events, particularly in post-PCI patients with CTO lesions.

## Data Availability

The dataset analyzed during the current study is available from the corresponding author on reasonable request.
